# Information Extraction from Clinical Texts with Generative Pre-trained Transformer Models

**DOI:** 10.7150/ijms.103332

**Published:** 2025-02-03

**Authors:** Min-Soo Kim, Philip Chung, Nima Aghaeepour, Namo Kim

**Affiliations:** 1 Department of Anesthesiology and Pain Medicine, Anesthesia and Pain Research Institute, Yonsei University College of Medicine, Seoul, Republic of Korea.; 2 Department of Anesthesiology, Perioperative and Pain Medicine, Stanford University School of Medicine, Stanford, California, USA.

**Keywords:** Natural Language Processing, Medical Records, Access to Information, Medical Informatics.

## Abstract

**Purpose:** Processing and analyzing clinical texts are challenging due to its unstructured nature. This study compared the performance of GPT (Generative Pre-trained Transformer)-3.5 and GPT-4 for extracting information from clinical text.

**Materials and Methods:** Three types of clinical texts, containing patient characteristics, medical history, and clinical test results extracted from case reports in open-access journals were utilized as input. Simple prompts containing queries for information extraction were then applied to both models using the Greedy Approach as the decoding strategy. When GPT models underperformed in certain tasks, we applied alternative decoding strategies or incorporated prompts with task-specific definitions. The outputs generated by GPT models were evaluated as True or False to determine the accuracy of information extraction.

**Results:** Clinical texts containing patient characteristics (60 texts), medical history (50 texts), and clinical test results (25 texts) were extracted from 60 case reports. GPT models could extract information accurately with simple prompts to extract straightforward information from clinical texts. Regarding sex, GPT-4 demonstrated a significantly higher accuracy rate (95%) compared to GPT-3.5 (70%). GPT-3.5 (78%) outperformed GPT-4 (57%) in extracting body mass index (BMI). Utilizing alternative decoding strategies to sex and BMI did not practically improve the performance of the two models. In GPT-4, the revised prompts, including definitions of each sex category or the BMI formula, rectified all incorrect responses regarding sex and BMI generated during the main workflow.

**Conclusion:** GPT models could perform adequately with simple prompts for extracting straightforward information. For complex tasks, incorporating task-specific definitions into the prompts is a suitable strategy than relying solely on simple prompts. Therefore, researchers and clinicians should use their expertise to create effective prompts and monitor LLM outcomes when extracting complex information from clinical texts.

## Introduction

An Electronic Health Record (EHR) is a digital repository of a patient's medical data, accessible electronically by healthcare providers 1. EHR data is categorized into structured and unstructured data based on the presence of a predetermined format 2. Clinical texts as unstructured data constitute a significant portion of medical data and contains valuable information 1, 3. However, processing and analyzing clinical texts is challenging due to the lack of a defined format and its narrative nature, posing significant obstacles to research based on clinical texts 1, 4.

Interest in large language models (LLMs) for clinical applications has surged within just a few months following the introduction of ChatGPT (Chat Generative Pre-trained Transformer; OpenAI), as conversational AI (artificial intelligence) application based on GPT models, a type of LLMs 5. A session of ChatGPT is initiated by entering a query, typically referred to as a “prompt”, and ChatGPT subsequently generates a natural language response 5. Natural language processing is a technology that transforms unstructured language into structured data 6. Thus, processing of unstructured clinical text may be possible by applying ChatGPT with the clinical text as input to a prompt containing a query for data processing.

Prompt engineering, such as using prompts with task-specific definitions, enables GPT models to effectively extract relevant information from unstructured clinical texts 7, 8. Additionally, selecting appropriate decoding strategies is crucial for improving performance in tasks with ambiguity or inconsistency 9, 10. Therefore, it is important to explore which approaches, including prompt engineering and decoding strategies, are most effective in addressing challenges when extracting information from clinical texts.

The pre-anesthesia evaluation note clearly summarizes the basic information, medical history, and test results of the surgical patients, making it useful for quickly understanding the patient's condition and assessing the patient's risk 11. The information in the pre-anesthesia evaluation note may be leveraged by clinicians as well as large language models to draft a range of medical documents, such as medical progress notes, surgical consent forms, and insurance claims 7, 12.

In this study, we compared the performance of GPT-3.5 and GPT-4 using prompts designed to extract information from unstructured clinical texts related to pre-anesthetic evaluations, derived from case reports published in open-access journals. Furthermore, we evaluated the effectiveness of alternative decoding strategies and the inclusion of task-specific definitions in prompts as methods to enhance performance in tasks where GPT models demonstrated limitations.

## Materials and Methods

### Data Sources and Clinical Texts as Input

We conducted a search for case reports in open-access journals related to anesthesiology through PubMed and obtained the original texts of 60 case reports that were published between 2018 and 2023. One author (MSK) extracted 60 clinical texts containing patient characteristics, 50 clinical texts containing patient's medical history, and 25 clinical texts containing clinical test results from the selected case reports.

### Designing Prompts for the GPT models

In this study, prompts for extracting information from clinical texts were designed based on zero-shot learning, a type of in-context learning where only task descriptions were provided without any examples 13.

The prompt for clinical texts containing patient characteristics contained a query that extracted basic information about patients undergoing surgery under anesthesia. The body mass index (BMI) was included in basic information extracted through the prompt, but a formula for calculating BMI using height and weight was not included in the prompt. This approach aimed to investigate whether GPT models could autonomously calculate BMI using height and weight values from clinical text without a BMI value, even in the absence of a BMI formula within the prompt. Thus, it was considered as a true response if the GPT models provided a BMI value from clinical texts with height and weight values but no BMI value. However, if GPT models did not provide BMI values despite the presence of height and weight values in clinical texts, it was specifically defined as a false response.

The prompt for clinical texts containing a patient's medical history was designed to ascertain the presence or absence of a specific disease, yielding a binary response. The prompt for clinical texts containing clinical test results were created with the aim of extracting information about the specific clinical test results.

### Extracting information using the GPT models

The GPT-3.5 and GPT-4 models were utilized through the OpenAI API (Application Programming Interface). This API offers a method for users to programmatically interact with the GPT models, allowing them to generate text, answer inquiries, carry out language-related tasks, and perform other functions 14. The created prompts were used to query GPT models through codes written using Python (version 3.8.13; Python Software Foundation, Wilmington, DE, USA), and LangChain (version 0.0.235; https://www.langchain.com) which is a Software framework for developing applications using large language models (LLMs). In the parameter setting of each model, 'gpt-3.5-turbo' and 'gpt-4' were set as models of GPT-3.5 and GPT-4, respectively. In the parameter settings of the ChatOpenAI Class imported from LangChain, the 'model' parameter was set to 'gpt-3.5-turbo' and 'gpt-4' to use the 'gpt-3.5-turbo-0613' and 'gpt-4-0613' models as GPT-3.5 and GPT-4, respectively. Each prompt was applied to both GPT-3.5 and GPT-4 only once to obtain a single output. As the main workflow, prompts containing queries to extract information were applied to GPT models using the Greedy Approach as the decoding strategy 10. To apply this strategy, the temperature parameter was set to zero to elicit a deterministic response, while default values were retained for all other parameters. Temperature controls the level of randomness in the output. When the temperature is set to 0, randomness is eliminated, and the model generates the consistent response for a given query 15.

After conducting the main workflow, if the performance of the GPT models for a specific task was low, the following process was performed to apply other decoding strategies instead of Greedy Approach. First, the same prompts used in the main workflow were applied to the GPT models five times with the temperature set to 1, resulting in five outputs. Self-Consistency, which determines the final output through majority voting for the five outputs, and First Valid Value, which selects the valid value as the final output if any of the five outputs have a valid value, were employed as decoding strategies 9. Self-Consistency has been proposed in previous research to complement the reasoning ability of LLMs 9. In programming, First Valid Value refers to selecting the first non-NA or non-NULL value from a list or vector. In this study, the concept of First Valid Value was applied as a decoding strategy. Despite the utilization of alternative decoding strategies, for tasks where GPT models demonstrated low performance, a revised prompt containing task-specific definitions was reapplied to both models.

### Evaluation of the generated outputs

Two authors (MSK and NK) proceeded with the evaluation of the output generated from GPT models. Each evaluator assessed the output as True for correct responses and False for incorrect responses. When the judgments between the two evaluators were different, the final judgment was determined through consensus.

In the case of clinical texts containing patient characteristics, if the GPT models failed to compute and provide BMI despite the presence of height and weight data, the response was considered incorrect. When only one of height or weight was present in the clinical texts where BMI existed, even if the GPT models were unable to calculate and provide the missing height or weight, it was not considered incorrect due to the complexity of the calculation involved. Regarding sex, if the GPT models were unable to confirm the sex despite the presence of clues such as sex-specific words or organs in the clinical text, the response was considered incorrect. Concerning surgery-related diagnosis, if the models provided a patient's diagnosis that wasn't directly linked to the surgery, it was considered an incorrect response.

### Embedding and dimension reduction of clinical texts

Sentence embedding was performed on clinical texts related to the low performance of GPT models for certain tasks, using the OpenAI API. The model utilized for embeddings was 'text-embedding-ada-002,' and the resulting output dimension after embeddings was 1536. After embedding, the 1536-dimensional real-number vectors obtained from each clinical text were reduced to 2-dimensional real-number vectors using t-Distributed Stochastic Neighbor Embedding (t-SNE) 16. The 2-dimensional vectors obtained through dimensionality reduction were labeled as true and false responses according to the responses of the GPT models for certain tasks with the low performance. The formation of clusters for each label was confirmed through visualization. The Silhouette score was calculated to provide a quantitative measure of how well-defined and distinct each cluster was 17. Dimensionality reduction using t-SNE as well as the calculation of the Silhouette score, was performed using scikit-learn library (version 1.3.0; https://scikit-learn.org/stable/). As a complementary approach, comparisons were conducted between true and false response for the mean values of the two components of the two-dimensional vectors obtained after dimensionality reduction. For these comparisons, Student's t test or Wilcoxon rank sum test was performed depending on the results of Shapiro-Wilk normality test for residuals derived from a linear regression model estimated from the mean values of two components and two labels.

### Statistical analysis

Statistical analyses and data visualization were conducted using R software (version 4.3.1; R Foundation for Statistical Computing). Data were presented as number or percentage. The comparison of the ratio of correct and incorrect responses between GPT-3.5 and GPT-4 was conducted using McNemar's test, with 95% confidence (CI) intervals of the *p* value derived from 1000 bootstrap samples to enhance reliability. When performing multiple comparisons of accuracy among the three decoding strategies, i.e., Greedy Approach, Self-Consistency, and First Valid Value, a Bonferroni correction was applied by multiplying the initially obtained *p* values by 3. A *p* value < 0.05 was considered statistically significant.

## Results

**Figure 1:**
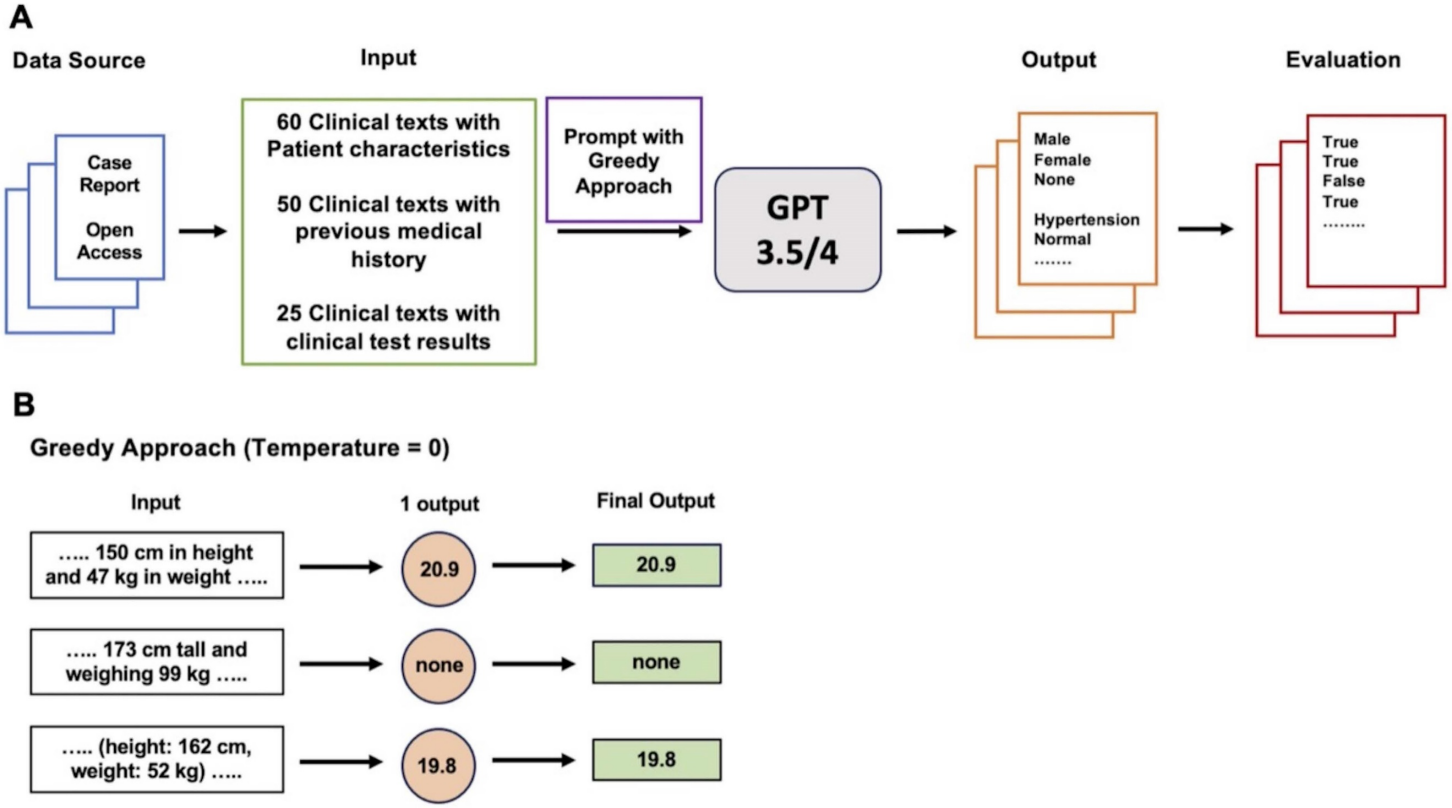
Main Workflow of this study.** A.** Three types of clinical texts extracted from case reports in open-access journals related to anesthesiology were utilized as input. Prompts containing queries for information extraction were then applied to both GPT-3.5 and GPT-4 using Greedy Approach as the decoding strategy. The resulting outputs were then assessed to determine the accuracy of information extraction. **B.** Greedy Approach. The temperature was set to zero so that GPT models could provide a consistent and deterministic response by eliminating randomness in the output.

The main workflow of this study was shown in Figure 1. The main workflow for collecting outputs generated from GPT models using prompts and codes was performed in August 2023. Further evaluations using alternative decoding strategies and the revised prompts were conducted in October 2023. Table 1 provides characteristics of sixty case reports enrolled in this study. All clinical texts with references are available in the Supplementary Data.

### Information extraction from clinical texts containing patient characteristics

**Figure 2:**
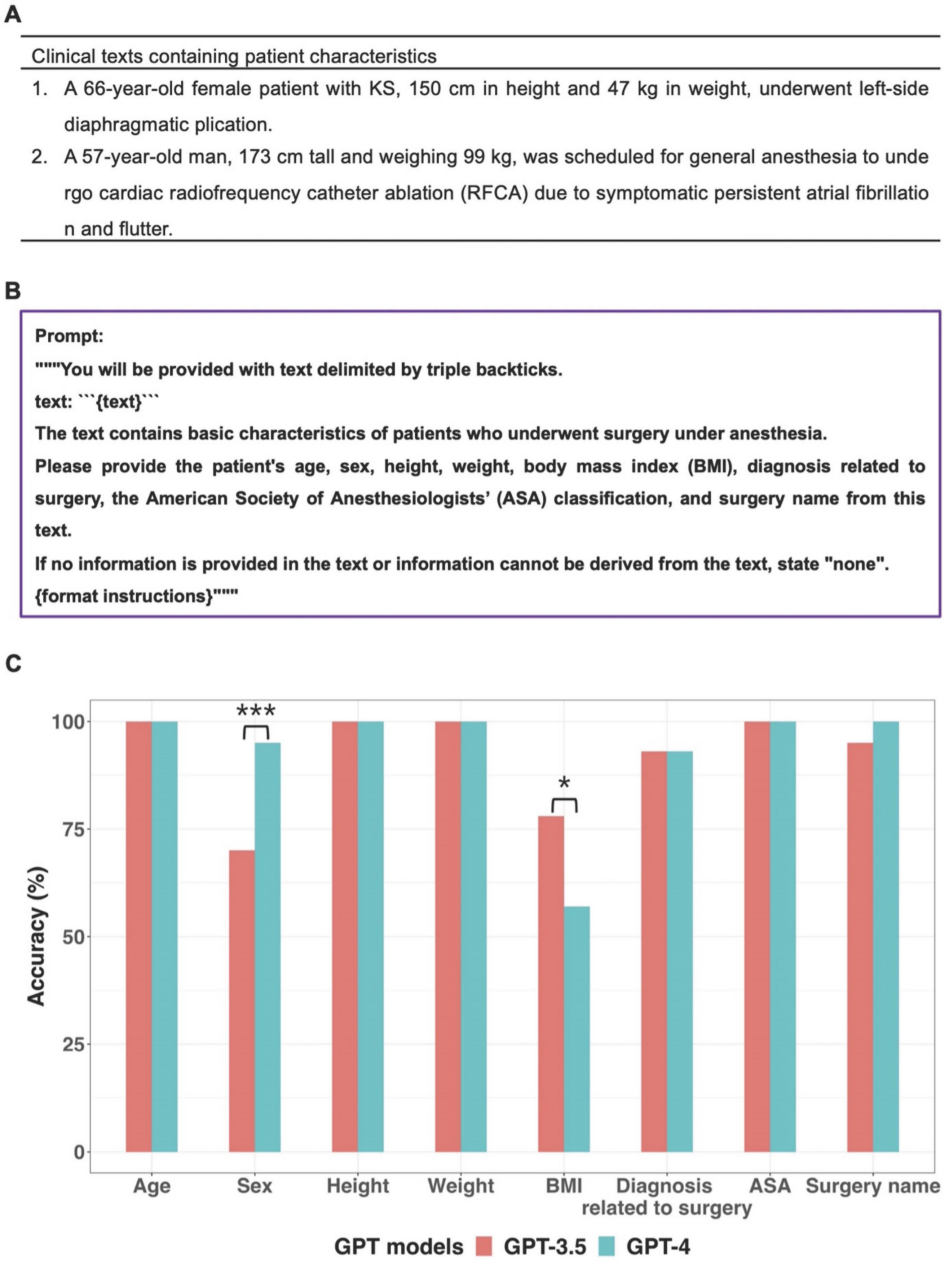
Information extraction from sixty clinical texts containing patient characteristics.** A.** Examples of clinical text containing patient characteristics.** B.** Prompt for information extraction. **C** GPT models' performance about extracting patient characteristics. In terms of sex, GPT-4 demonstrated a significantly higher accuracy rate (95%) compared to GPT-3.5 (70%). GPT-3.5 exhibited significantly better performance (78%) for BMI extraction compared to GPT-4 (57%). *p value < 0.05, **p value < 0.01, ***p value < 0.001.

Examples of clinical text containing patient characteristics, the prompt and GPT models' performance for extracting patient characteristics with Greedy Approach were presented in Figure 2. Both models accurately extracted data regarding age, height, weight, and ASA (American Society of Anesthesiologists) classification from all clinical texts. For surgery name, only GPT-4 demonstrated a 100% accuracy rate without statistically significant difference between the two models (*p* = 0.248, 95% CI: 0.023 to 1.000).

Regarding sex, neither of the two models achieved a 100% accuracy rate, but GPT-4 demonstrated a statistically significantly higher accuracy rate (95%) compared to GPT-3.5 (70%) (*p* < 0.001, 95% CI: 7.6 × 10^-6^ to 0.043). For the eighteen clinical texts where GPT-3.5 provided incorrect sex information, it consistently misclassified “male” as “none.” This misclassification was due to the inability of GPT-3.5 to identify “man,” “boy,” and “he” as male-related words, as well as “prostate” as male-related organs.

**Figure 3:**
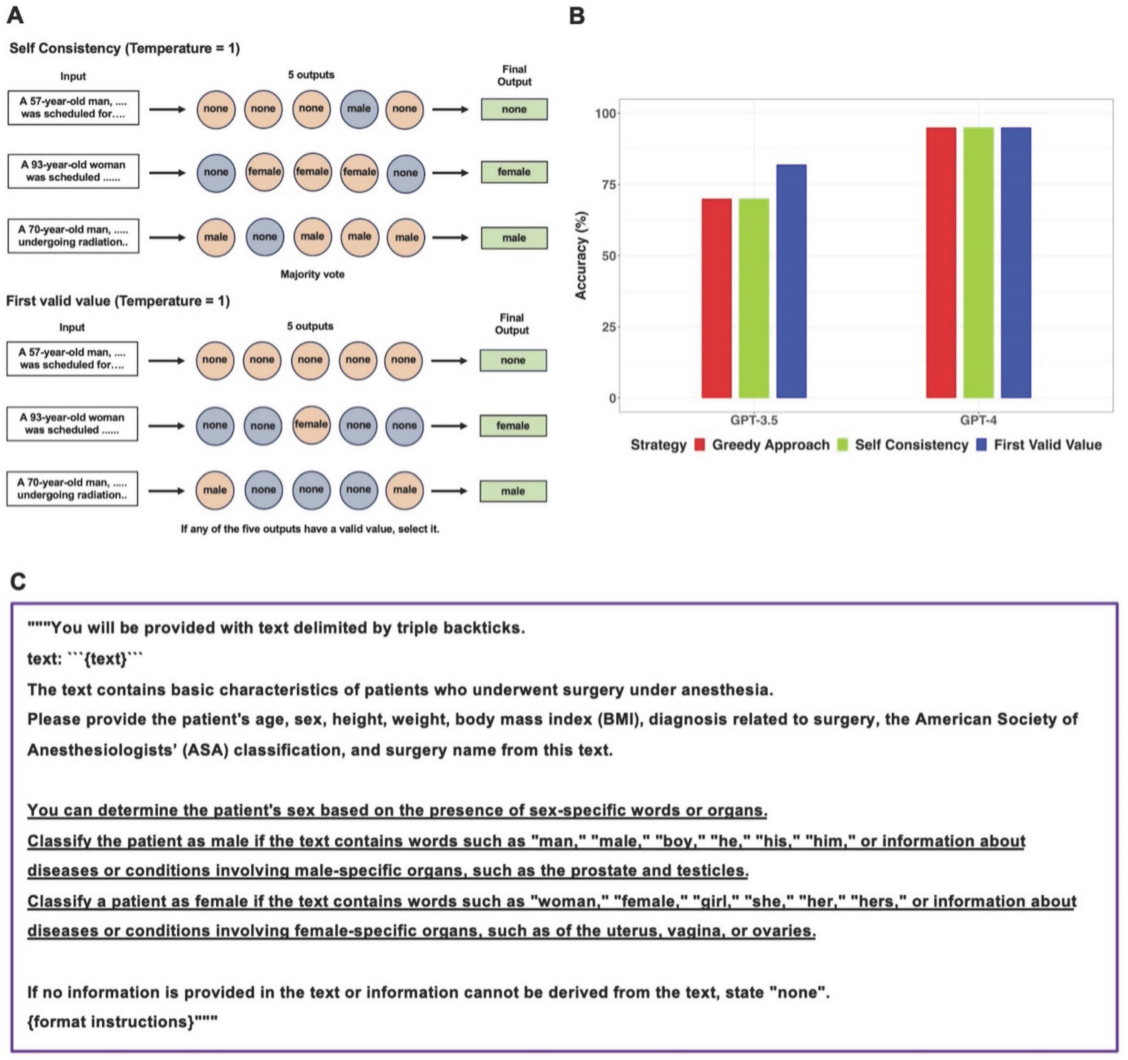
Further evaluations for sex information extraction from sixty clinical texts using alternative decoding strategies.** A.** The original prompt used in the main workflow was applied to the GPT models five times with the temperature set to 1, resulting in five outputs. Self-Consistency, which determines the final output through majority voting for the five outputs, and First Valid Value, which selects the final output if any of the five outputs have a valid value instead of “none”, were employed as alternative decoding strategies. **B.** The performance of GPT models in extracting sex information from sixty clinical texts was evaluated using three different strategies. There were no significant differences among the three decoding strategies in both models. **C.** The revised prompt was created by adding sex-related clues to the original prompt. In GPT-3.5, the revised prompt corrected 17 out of 18 cases of incorrect sex identification. In GPT-4, the revised prompt successfully corrected all three previously incorrect cases.

Specifically, in 14 out of 16 clinical texts that contained the word “man”, GPT-3.5 categorized the sex as “none”. GPT-4 provided incorrect sex information in three clinical texts. In one case, GPT-4 failed to identify the word “woman,” and in the other two cases, it missed recognizing the words “he” and “prostate.” When the GPT models generated “male” or “female” instead of “none”, those answers were always correct. Performance of GPT models in extracting sex from sixty clinical texts was additionally evaluated using Self Consistency and First Valid Value as alternative decoding strategies. Schematic diagrams of these alternative strategies used for further evaluations were shown in Figure 3A. The results of further evaluations were summarized in Figure 3B. In GPT-3.5, despite First Valid Value (82%) providing better accuracy than Greedy Approach (70%) and Self Consistency (70%), there was no statistical difference (*p* = 0.023; 95% CI: 0.0015 to 0.248; Bonferroni corrected *p* = 0.070). In GPT-4, alternative decoding strategies did not contribute to performance improvement. A revised prompt containing definitions of each sex category (Figure 3C) was employed using the Greedy Approach on clinical texts from which incorrect sex information had been extracted when the original prompt was used in both models. In GPT-3.5, 17 out of 18 cases that previously resulted in incorrect sex identification were corrected to display the accurate sex response with the revised prompt. GPT-3.5 still exhibited an inability to identify the word “he” correctly. Upon executing the revised prompt in GPT-4, the three cases that had previously generated incorrect responses were rectified to provide the accurate answers.

**Figure 4:**
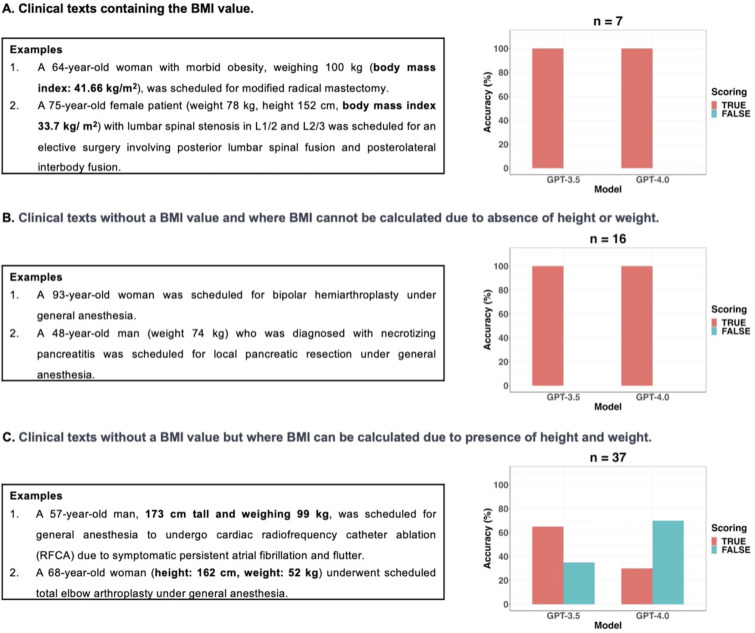
Three types of clinical texts according to the presence or absence of BMI values and the possibility of calculating BMI using height and weight.** A.** There were no false responses for BMI value extraction from the two GPT models in clinical texts with BMI values. **B.** There were no false responses for BMI value extraction from the two GPT models in clinical texts without BMI and where BMI cannot be calculated due to absence of height or weight. **C.** If GPT models did not provide BMI values despite the presence of height and weight values in the clinical texts without BMI values, these instances were considered as false responses in this study. All false responses in BMI extraction occurred only in the thirty-seven clinical texts without a BMI value but with height and weight information, allowing BMI calculation.

**Figure 5:**
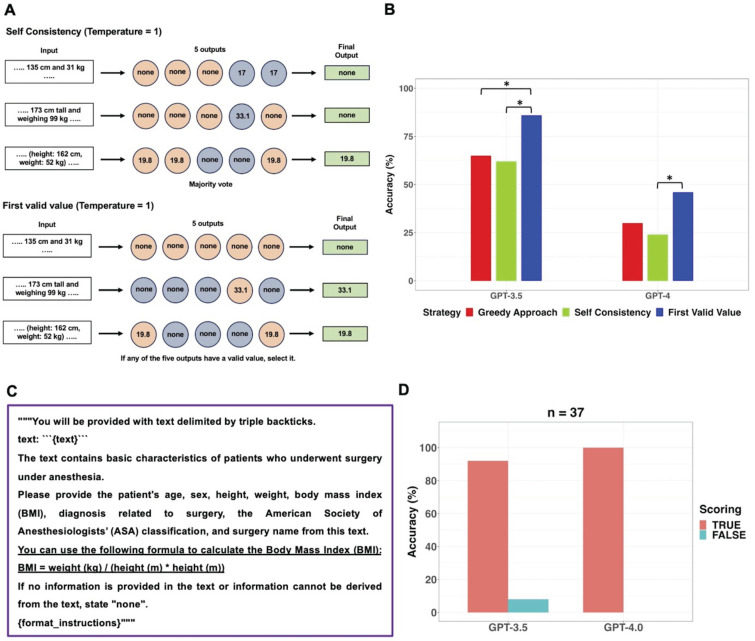
Further evaluations for BMI extraction from thirty-seven clinical texts containing height and weight but no BMI values using alternative decoding strategies and revision of the prompt.** A.** The original prompt used in the main workflow was applied to the GPT models five times with the temperature set to 1, resulting in five outputs. Self-Consistency, which determines the final output through majority voting for the five outputs, and First Valid Value, which selects the final output if any of the five outputs have a valid value instead of “none”, were employed as alternative decoding strategies. **B.** The performance of GPT models in extracting BMI from the thirty-seven clinical texts, which contained height and weight values but no BMI values, was evaluated using three different strategies. In GPT-3.5, the accuracy of First Valid Value (86%) was statistically significantly higher than that of Greedy Approach (65%) and Self Consistency (62%), respectively. In GPT-4, the accuracy of First Valid Value (46%) was statistically significantly higher than that of Self Consistency (24%). **C.** The revised prompt was created by adding the BMI formula to the original prompt. **D.** Upon implementing the revised prompt through the Greedy Approach, GPT-4 answered with 100% accuracy, GPT-3.5 persisted in failing to generate BMI values in three clinical texts. *adjusted *p* value < 0.05, **adjusted *p* value < 0.01, *** adjusted *p* value < 0.001.

GPT-3.5 exhibited statistically significant better information extraction performance (78%) in BMI compared to GPT-4 (57%) (*p* = 0.012; 95% CI: 6.1 × 10^-5^ to 0.503). According to the presence or absence of BMI values and the possibility of calculating BMI using height and weight, clinical texts were categorized into three types: seven clinical texts containing the BMI value, sixteen clinical texts without a BMI value and where BMI cannot be calculated due to absence of height or weight, and thirty-seven clinical texts without a BMI value but where BMI can be calculated due to presence of height and weight values. In this study, if GPT models did not provide BMI values despite the presence of height and weight values in clinical texts without a BMI value, it was considered as a false response. Figure 4 displayed examples of three types of clinical texts and the performance of GPT models in extracting BMI values based on the type of clinical text. All false responses in BMI extraction occurred only in the thirty-seven clinical texts without a BMI value but with height and weight information, allowing BMI calculation (Figure 4C). In these thirty-seven clinical texts, GPT-3.5 and GPT-4.0 provided BMI values as the true response using height and weight values in 24 and 11 cases, respectively. When the GPT models generated BMI values instead of “none”, those answers were always correct. Thus, the performance of GPT models in extracting BMI from only thirty-seven clinical texts of this type was further evaluated using Self Consistency and First Valid Value as alternative decoding strategies. Schematic diagrams of these alternative strategies used for further evaluations were shown in Figure 5A.

Results of further evaluation of the GPT models in extracting BMI from thirty-seven clinical texts are presented in Figure 5B. In GPT-3.5, the accuracy of First Valid Value (86%) was statistically significantly higher than that of Greedy Approach (65%; *p* = 0.013; 95% CI: 8.7 × 10^-4^ to 0.136; Bonferroni corrected *p* = 0.040) and Self Consistency (62%; *p* = 0.008; 95% CI: 5.1 × 10^-4^ to 0.134; Bonferroni corrected *p* = 0.023), respectively. In GPT-4, the accuracy of First Valid Value (46%) was statistically significantly higher than that of Self Consistency (24%; *p* = 0.013; 95% CI: 8.7 × 10^-4^ to 0.248; Bonferroni corrected *p* = 0.040). Instead of altering the decoding strategy, the performance of the GPT models for BMI extraction was assessed by revising the prompt within the existing main workflow with Greedy Approach. The prompt revision involved incorporating the BMI formula, which defines BMI, into the original prompt (Figure 5C). Upon implementing the revised prompt, which included the BMI formula, through the Greedy Approach, GPT-4 answered with 100% accuracy. However, GPT-3.5 persisted in failing to generate BMI values in three instances of clinical texts containing height and weight values but no BMI values (Figure 5D).

**Figure 6:**
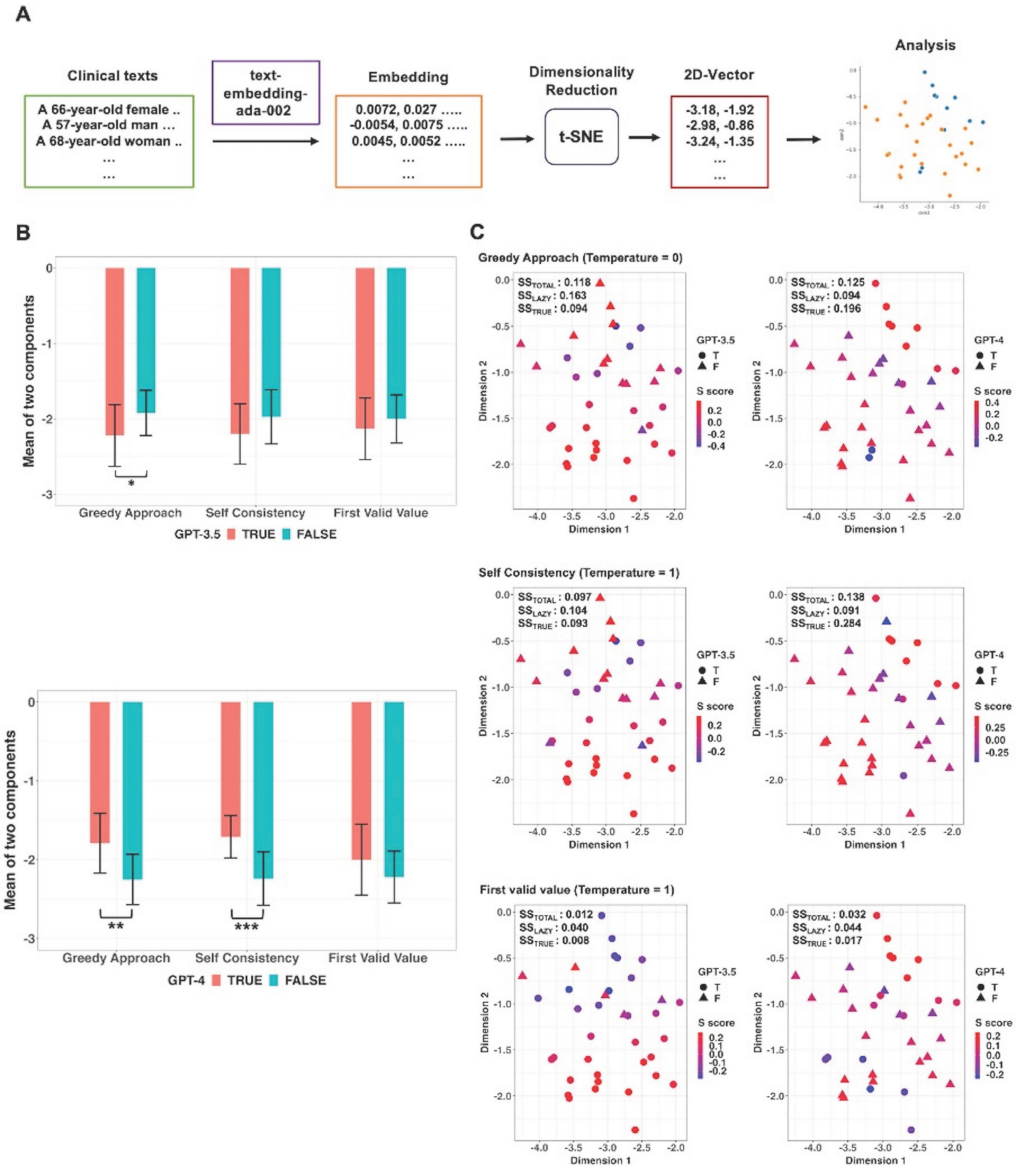
Embedding and dimensionality reduction of thirty-seven clinical texts containing height and weight but no BMI.** A.** Sentence embedding was performed on clinical texts and then real-number vectors obtained from each clinical text were reduced to two-dimensional vectors using t-Distributed Stochastic Neighbor Embedding (t-SNE). The two-dimensional vectors were labeled as true and false responses based on the GPT models' responses. **B.** According to decoding strategies, comparisons were conducted between the two responses for the mean values of the two components of the two-dimensional vectors. There was a significant difference in mean values between the true and false responses with the Greedy Approach in GPT-3.5 and both the Greedy Approach and Self Consistency in GPT-4. **C.** Scatter plots with Silhouette score of true and false responses according to the three kinds of strategies. *adjusted *p* value < 0.05, **adjusted *p* value < 0.01, *** adjusted *p* value < 0.001.

Two-dimensional vectors were obtained through dimensionality reduction using t-SNE from the embedding vectors of the thirty-seven clinical texts containing height and weight values but no BMI values and additional analyses were conducted in these vectors (Figure 6A). The mean values of the two components of the two-dimensional vectors were compared between True and False responses generated from the GPT models using original prompt without the BMI formula according to each decoding strategy (Figure 6B). In GPT-3.5, there was a statistically significant difference in mean values of two components between True and False responses in the Greedy Approach (*p* = 0.015; 95% CI: 9.2 × 10^-6^ to 0.435; Bonferroni corrected *p* = 0.045). In GPT-4, statistically significant differences in mean values of the two elements were observed between True and False responses in Greedy Approach (*p* = 0.003; 95% CI: 3.3 × 10^-11^ to 0.335; Bonferroni corrected *p* = 0.009) and Self Consistency (*p* < 0.001; 95% CI: 6.4 × 10^-12^ to 0.092; Bonferroni corrected *p* < 0.001), respectively. Scatter plots and Silhouette scores of True and False responses of the GPT models according to three decoding strategies were illustrated in Figure 6C. In GPT-4, mean values of Silhouette score for the vectors corresponding to the True response under Greedy Approach and Self Consistency were 0.196 and 0.284, respectively.

### Information extraction from clinical texts containing previous medical history

**Figure 7:**
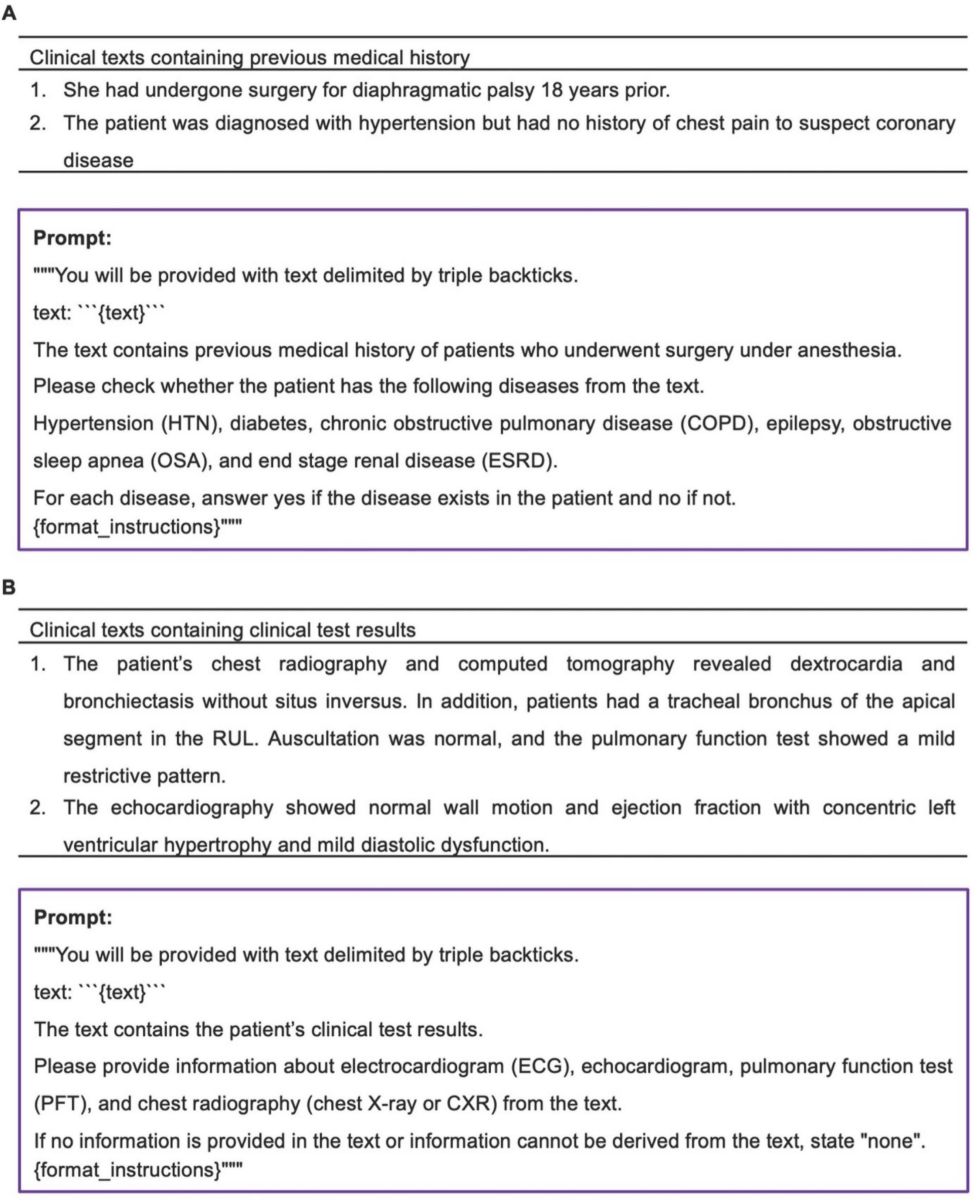
Information extraction from clinical texts containing previous medical history or clinical test results.** A.** Examples of clinical texts containing previous medical history and prompt for verifying the presence of a specific disease. **B.** Examples of clinical texts containing clinical text results and prompt for extracting clinical test results.

When using the prompt to verify the presence of a specific disease in a clinical text containing a patient's medical history (Figure 7A), GPT-4 accurately confirmed the presence or absence of the disease with 100% accuracy in all instances. In the case of GPT-3.5, the presence or absence of a specific disease was accurately determined, except for one instance where chronic renal failure was mistakenly classified as end stage renal disease (ESRD).

### Information extraction from clinical texts containing clinical test results

Examples of clinical texts containing clinical text results and prompt for extracting clinical test results were illustrated in Figure 7B. In both models, there was a single instance of incorrect information extraction from a chest X-ray when utilizing the prompt designed to extract specific test information from clinical text containing clinical test results. In this isolated case, both models interpreted the findings of chest computerized tomography as those of a chest X-ray. Aside from this particular occurrence, both models consistently and accurately extracted clinical test results from all clinical texts.

## Discussion

In this study, both models accurately extracted data regarding age, height, weight, and ASA classification from all clinical texts containing patient characteristics. Regarding sex, GPT-4 demonstrated a significantly higher accuracy rate (95%) compared to GPT-3.5 (70%). However, GPT-3.5 exhibited statistically significantly better performance (78%) for extracting BMI compared to GPT-4 (57%). In GPT-4, all incorrect responses regarding sex and BMI during the main workflow were rectified by revising the original prompt to include definitions of each sex category or the BMI formula. Despite applying the revised prompts, GPT-3.5 failed to answer with 100% accuracy for extracting Sex or BMI. Utilizing alternative decoding strategies for sex and BMI did not practically improve the performance of the two models. Additionally, both models demonstrated high accuracy in identifying specific diseases and extracting specific test results.

Accurate sex identification is essential due to the inherent differences in anatomy, disease prevalence, and prognosis according to a patient's sex 18, 19. In this study, GPT-3.5 did not demonstrate satisfactory performance in accurately extracting sex from clinical texts. Especially, GPT-3.5's inability to identify the word 'man' as male in many texts could be due to the various dictionary meanings of “man”. In traditional usage, the word "man" is occasionally employed to denote humanity as a whole 20. This broader semantic scope may account for GPT-3.5's challenges in accurately discerning sex, as it potentially interpreted "man" in its more universal sense. A possible example of such situations in clinical text can be found in abbreviations for diseases or medications. For instance, if the abbreviation "MS" is mentioned in clinical texts, it could represent several different meanings (e.g., mitral stenosis, multiple sclerosis, or magnesium sulfate), necessitating contextual interpretation. In texts that contain polysemous terms like this, an incorrect interpretation by the LLMs can lead to the extraction of erroneous information. Both models also failed to identify male in a clinical text that mentioned the prostate, an organ exclusive to males. In this study, these limitations could be significantly reduced by incorporating definitions of each sex category directly into the prompts. Therefore, if researchers or clinicians provide clear definitions of the target variables they wish to extract in the prompts, utilizing their own domain knowledge rather than relying solely on LLMs, it could enhance LLM performance.

In this study, the use of a prompt without a BMI formula aimed to verify the GPT models' capability to comprehend the formula of BMI, and subsequently calculate and provide BMI using height and weight data extracted from clinical texts without BMI information. Both GPT models successfully generated BMI in some clinical texts when provided with height and weight data. It is believed that the GPT models possess prior knowledge of BMI, enabling them to calculate BMI using values of height and weight. However, both GPT models failed to generate BMI in all clinical texts where height and weight values were present but not BMI values. In a previous study on clinical text summarization utilizing LLMs, GPT-4 tended to adopt a literal approach without interpretation, in contrast of medical experts 21. This tendency may have led to GPT models in the current study failing to interpret the prompt broadly enough to calculate BMI from height and weight in some of the clinical texts. The authors of this previous study noted the need to investigate whether the model responds literally or more interpretively through temperature control 21. Despite setting the temperature to 1 and employing the Self-Consistency method in this study, the models' performance in extracting BMI information did not improve. The inconsistency in generating BMI may also stem from variations in contextual differences and the use of specialized medical terminology within clinical texts containing height and weight information 1, 22. To find clues related to this hypothesis, this study conducted additional analyses using sentence embedding 23. Sentence embedding refers to a numeric representation of a sentence in the form of a vector of real numbers, which encodes meaningful semantic information such as context and meaning 24. By applying t-SNE to these high-dimensional vectors, we were able to project each clinical text onto a two-dimensional plane, enabling a visual inspection of clusters based on true and false responses generated by GPT models. In addition to visual evaluation, this study calculated Silhouette scores to quantify the cohesion of clusters. The Silhouette score ranges from -1 to 1, with a value closer to 1 indicating that the clusters are well-formed and internally cohesive 17. In this study, Silhouette scores ranging from 0 to 0.15 were calculated, indicating that the clusters were not well separated and there was overlap between them. The low Silhouette scores might be attributed to several factors, including the possibility that the t-SNE may not adequately preserve the original data structure, the limited number of clinical texts, which may not be sufficient to form distinct and cohesive clusters, and the potential for GPT models to randomly decide whether to calculate BMI values 16. Further research is warranted to address these issues.

Interestingly, GPT-3.5 demonstrated the ability to generate BMI from a greater number of clinical texts compared to GPT-4 when using the prompt without a BMI formula. These results indicate that GPT-4 is more advanced in certain aspects, but it might not be optimized for specific types of data processing 25. Both GPT-3.5 and GPT-4 utilize the "cl100k_base" tokenizer, which is based on Byte Pair Encoding (BPE) 26. Although the tokenization process is consistent across the two GPT models, differences may arise from their training datasets such as variations in content and coverage and training procedures, including hyperparameters, schedules, and fine-tuning stages 27. These differences may result in distinct model biases, potentially making GPT-4 less effective than GPT-3.5 for certain tasks. Another possible explanation for GPT-3.5's outperformance could be the trade-off between hallucination and creativity. Hallucination, defined as a false response generated by LLMs, can be seriously dangerous in medical situations 7. However, a previous study suggested that the creativity of GPT models could be enhanced by the phenomenon of hallucination by allowing them to investigate a broader range of token sequences beyond just the most likely options on the based on the given input 28. A recent study showed that GPT-4 exhibited fewer hallucinations than GPT-3.5 29. Although reducing hallucinations improves factual accuracy, it may inadvertently limit GPT-4's creativity. In other words, this trade-off could lead GPT-4 to generate more conservative interpretations of queries and responses. Consequently, GPT-4 may have calculated and provided BMI information less frequently in clinical texts containing height and weight. Therefore, regardless of how advanced an LLM may be, continuous monitoring will be required to ensure that it accurately extracts the specific outcomes that researchers or clinicians seek from clinical texts.

In this study, Self-Consistency and First Valid Value were applied as alternative decoding strategies to improve the low performance of sex and BMI extraction of the GPT models. Our findings suggested that Self-Consistency offered negligible performance enhancement for GPT models, due to these advanced language models' inherent efficiency in extracting information from input prompts. In this study, when the GPT models provided sex and BMI instead of “none”, those values were always accurate. So, the primary issue with extracting such information is not that GPT models generate incorrect values, but whether they can utilize or interpret the data present in clinical texts to provide values. Thus, if any of the five outputs obtained by applying the original prompt five times to the GPT models contained a value other than “none”, selecting an output with that value as the final output could enhance performance. In this study, the use of First Valid Value as a decoding strategy demonstrated better performance for extracting BMI from the thirty-seven clinical texts containing height and weight values but no BMI values in GPT-3.5, compared to Greedy Approach and Self-Consistency. However, it is essential to verify that the outputs provided by the LLM are accurate in order to apply the First Valid Value.

This study showed that the prompt with a BMI formula as a definition in the form of a mathematical formula led to significant performance improvements in both models for providing BMI. In particular, the performance of GPT-4 was greatly improved with 100% accuracy in generating BMI. In healthcare, it is common to utilize existing patient information to create clinically significant scoring systems such as Child Pugh Score and Model for End-Stage Liver Disease (MELD) in liver disease 30, 31. Thus, it may be necessary to provide detailed definitions about each score system in the prompt when generating these scores from clinical texts through LLMs. In addition, embedding documents that detail scoring systems, combined with Retrieval-augmented generation (RAG), could be considered an effective way to provide prompts with task-specific information 32.

The current study had some limitations. First, this study demonstrated the potential of GPT models in extracting information from well-structured clinical texts in case reports. However, real-world clinical texts derived from EHRs are typically written in an informative-dense and telegraphic style and are often more fragmented and prone to errors, which presents challenges for data processing and analysis 1. Second, the relatively small number of case reports enrolled in this study may have constrained the depth and comprehensiveness of the evaluation. This limitation could also potentially increase the risk of overfitting and limit the generalizability of the findings to other clinical texts. Despite the limitations posed by the structured nature of the clinical texts and small sample size, the insights regarding prompt engineering for extracting information from clinical texts derived from our study may be applied to improve model performance in real-world clinical settings. In this study, application of bootstrap resampling helped mitigate statistical issues associated with a small dataset by producing a synthetic dataset that is representative of the broader population of clinical case reports, thereby enabling better estimation of the population confidence intervals 33. We also employed Bonferroni correction to conservatively adjust the significance level in our statistical hypothesis testing to ensure that our findings were not false-positive discoveries on a small dataset 34. Third, clinical texts can potentially contain protected health information (PHI) 35. Until safer methods for conducting research using LLMs are available, it is crucial to take proactive measures to prevent risks to patient privacy and potential harm. Thus, we carefully considered the ethical concerns associated with AI research using clinical texts. To adhere to ethical standards and mitigate any potential risks to patient privacy, we utilized clinical texts from case reports published in open-access journals, which are publicly available and contain no PHI. Future research on EHR data using LLMs may require strategies to safeguard patient PHI, such as de-identifying data, deploying LLMs in privacy-preserving local environments, or collaborating with healthcare institutions equipped with secure analytical infrastructure 36. Fourth, this study focused solely on performance comparisons among GPT models and did not include other LLMs, such as clinical domain-specific models like BioBERT or ClinicalBERT. While comparing clinical domain-specific models for clinical information extraction would be valuable, these models are less accessible to clinical practitioners compared to GPT models 7. Additionally, we prioritized examining the utility and challenges of alternative decoding strategies and prompt engineering when extracting information from clinical text using GPT models. To align with this objective, we concentrated on comparisons within a single model type rather than across multiple models. In contrast to other studies 9, we found that alternative decoding strategies did not significantly improve LLM task performance in extracting extraction from clinical vignettes. However, prompt augmentation using task-specific definitions for the element being extracted showed significant performance benefit. This technique can be readily applied in future studies by healthcare practitioners for a variety of information extraction tasks in healthcare. Finally, while prompts incorporating task-specific definitions can improve performance, they may not fully address the intrinsic limitations of the models. Given the rapidly evolving field of LLM technology, further evaluation of their information extraction capabilities using real EHR data will remain essential in clinical settings to ensure the accurate and objective extraction and interpretation of information, free from hallucinations 7, 29. Prompt engineering is an evolving field, with techniques likely to change as newer models are introduced. Thus, this research highlights one of many possible methods to improve performance. In such contexts, applying optimized prompts and continuously monitoring outcomes obtained from LLMs to refine them will be essential for improving performance and ensuring reliable results in clinical settings.

In summary, GPT models could perform adequately with simple prompts for extracting straightforward information from clinical texts. However, for more complex tasks, where GPT models may need to utilize their prior knowledge or context-based interpretation, incorporating task-specific definitions in the prompt could be the suitable strategy rather than relying solely on simple prompts. Therefore, when extracting complex information from clinical text using LLMs, researchers and clinicians should actively use their domain knowledge to craft effective prompts and continuously monitor the accuracy of the outcomes provided by the LLMs.

## Supplementary Material

Supplementary tables.

## Author Contributions

**Min-Soo Kim:** Conceptualization, Methodology, Software, Formal analysis, Investigation, Resources, Data curation, Visualization, Writing - original draft, Writing - Review & Editing, Project Administration.

**Namo Kim:** Investigation, Methodology, Resources, Writing - Review & Editing.

**Philip Chung:** Writing - review & editing.

**Nima Aghaeepour:** Conceptualization, Methodology, Writing - review & editing, Supervision, Project Administration.

## Figures and Tables

**Table 1:** Characteristics of sixty case reports

		n = 60
Age (yr)		59 [0.17 - 93]*
Patient Category		
	Adult	51 (85)
	Pediatric	9 (15)
Sex		
	Male	36 (60)
	Female	24 (24)
Author Country of Origin		
	Asia	53 (88.3)
	Europe	3 (5)
	North America	3 (5)
	Africa	1 (1.7)
Publication year		
	2018	1 (1.7)
	2019	15 (25)
	2020	9 (15)
	2021	10 (16.7)
	2022	15 (25)
	2023	10 (16.7)

Data are given as median [range] or number (%). *One case described the patient's age as 'middle-aged,' which was approximated as 50 years to calculate the median and range.

## Abbreviations

GPT: generative pre-trained transformer
BMI: body mass index
EHR: electronic health record
LLMs: large language models
ChatGPT: chat generative pre-trained transformer
AI: artificial intelligence
API: application programming interface
NA: not applicable
t-SNE: t-distributed stochastic neighbor embedding
ASA: American Society of Anesthesiologists
HTN: hypertension
COPD: chronic obstructive pulmonary disease
OSA: obstructive sleep apnea
ESRD: end stage renal disease
ECG: electrocardiogram
PFT: pulmonary function test
CXR: chest X-ray
MELD: end-stage liver disease
RAG: retrieval-augmented generation
PHI: protected health information
