# Supplementary material for: Information Extraction from Clinical Texts with Generative Pre-trained Transformer Models
Source: Int J Med Sci. 2025 Feb 3;22(5):1015–28. doi: 10.7150/ijms.103332 (PMC11866537; doi:10.7150/ijms.103332)
Supplement: Supplementary file 1 — Supplementary tables. [file ijmsv22p1015s1.pdf]

Table S1. Clinical text for patient characteristics.

1. A 66-year-old female patient with KS, 150 cm in height and 47 kg in weight, underwent left-side diaphragmatic plication [1].
2. A 57-year-old man, 173 cm tall and weighing 99 kg, was scheduled for general anesthesia to undergo cardiac radiofrequency catheter ablation (RFCA) due to symptomatic persistent atrial fibrillation and flutter [2].
3. A 68-year-old woman (height: 162 cm, weight: 52 kg) underwent scheduled total elbow arthroplasty under general anesthesia [3].
4. A 60-year-old woman diagnosed with a distal anterior cerebral artery aneurysm was scheduled for a craniotomy and clipping of the aneurysm [4].
5. A 93-year-old woman was scheduled for bipolar hemiarthroplasty under general anesthesia [5].
6. A 73-year-old woman (height: 154 cm, weight: 55 kg) was diagnosed with a malignant lung nodule in the left lower lobe that was incidentally discovered on computed tomography. The patient underwent video-assisted thoracoscopic surgery for left lower lobectomy [6].
7. A 48-year-old man (weight 74 kg) who was diagnosed with necrotizing pancreatitis was scheduled for local pancreatic resection under general anesthesia [7].
8. A 44-year-old male patient (weight 60 kg) was posted for C2–C5 astrocytoma excision under general anesthesia [8].
9. A 75-year-old woman with well-controlled hypertension and diabetes mellitus (height: 154.1 cm, weight: 52.8 kg) was scheduled to undergo a transforaminal lumbar interbody fusion of L3-S1 [9].
10. A 64-year-old woman with morbid obesity, weighing 100 kg (body mass index: 41.66 kg/m2), was scheduled for modified radical mastectomy [10].
11. A 75-year-old female patient (weight 78 kg, height 152 cm, body mass index 33.7 kg/ m2) with lumbar spinal stenosis in L1/2 and L2/3 was scheduled for an elective surgery involving posterior lumbar spinal fusion and posterolateral interbody fusion [11].
12. A 60-year-old male (weight: 64 kg, height: 159 cm) was scheduled for emergency incision, drainage, and flap coverage on soft tissue infection of the right hand [12].
13. A 78-year-old female patient (height: 150 cm; weight: 40 kg) was hospitalized for breast cancer and scheduled for hook-wire excision and sentinel lymph node biopsy. Her physical status was classified as the American Society of Anesthesiologists III [13].

14. A 78-year-old male with a history of coronary artery disease, angina pectoris, and hypercholesterolemia was diagnosed with a cT3N2M0 adenocarcinoma of the distal esophagus. After neo-adjuvant chemoradiation (41.4 Gy in 23 fractions with 5 weeks of Carboplatin and Paclitaxel), a minimally invasive transthoracic esophagectomy according to Ivor Lewis was scheduled [14].
15. A 70-year-old man (weight 70 kg, body mass index 25.7 kg/m2) with underlying hypertension and dyslipidemia underwent an elective open hernioplasty for right inguinal hernia under ambulatory surgery [15].
16. A 10 year-old boy with a compound fracture of the tibia was scheduled for an emergent external fixator application [16].
17. A 35-year-old woman, weighing 65 kg, who had a C2–C3 intradural extramedullary meningioma with no known comorbidities or history of previous surgeries was indicated for laminectomy and tumor excision [17].
18. He was a 42-year-old man, weighing 160 kg, BMI 54 kg/m2, scheduled for total thyroidectomy and bilateral neck dissection for metastatic papillary thyroid cancer with paratracheal lymph node and left recurrent laryngeal nerve involvement [18].
19. A 42-year-old man (weight: 78 kg, height: 175 cm) underwent an elective functional endoscopic sinus surgery and revision of septoplasty under general anesthesia [19].
20. A 35-year-old man (weight, 80 kg; height, 165 cm) with chronic persistent left hip pain, without any known comorbidities, was diagnosed with avascular necrosis of the femoral head of the left hip joint and was scheduled for uncemented left hip total arthroplasty [20].
21. A 42-year-old woman underwent a hysteroscopic resection of uterine fibroids under general anesthesia with informed consent [21].
22. A 4-year-old female patient (weight 15 kg, American Society of Anesthesiologists physical status I) was scheduled for surgical treatment of developmental dysplasia of the right hip under general anesthesia [22].
23. A 77-year-old man, who was 172 cm tall and weighed 70 kg, was scheduled for radical mastectomy and axillary clearance for breast cancer [23].
24. A 17-year-old man, with a height of 168 cm and weight of 55 kg, was admitted to the hospital to undergo a wide excision and reconstruction with tumor prosthesis for an osteosarcoma of the right distal femur [24].

25. A 34-year-old man (weight: 79 kg, height: 180 cm), who underwent removal of an oligodendroglioma in the left temporal lobe 4 years ago, was admitted for the removal of the recurrent tumor. An awake craniotomy was planned [25].
26. A 70-year-old man (168 cm, 72.4 kg) with right upper lobe lung cancer was scheduled to undergo right upper lobectomy with four-port VATS (two each in the fourth and seventh intercostal spaces) [26].
27. A 24-month-old male patient with a height of 87 cm and a weight of 10.5 kg presented to the emergency room due to respiratory failure, mental decrease to a stupor state, systemic cyanosis, and lactic acid increase up to 12.3 mM/L (normal range: 0.7–2.5 mM/L) [27].
28. An 80-year-old female (156 cm, 60 kg) with American Society of Anesthesiologists physical status classification IV required emergency V-P shunt surgery for her progressing hydrocephalus [28].
29. A 74-year-old female patient was scheduled to undergo cranioplasty and removal of a meningioma under general anesthesia [29].
30. A 33-year-old male (60 kg) diagnosed with type 3b kyphosis of the thoracic region at the L1 level underwent extended pedicle subtraction osteotomy [30].
31. A 39-year-old male patient with no known comorbidities or allergies had a closed, complete, displaced fracture in the middle third shaft of the right clavicle due to a history of trauma [31].
32. A 27-year-old parturient woman with a 31-week gestational age underwent cesarean delivery under combined spinal-epidural anesthesia. She weighed 60 kg and was 165 cm tall [32].
33. A 70-year-old male (height 154.6 cm, weight 53.6 kg) was diagnosed with hepatic cell carcinoma and admitted for laparoscopic right colectomy posterior sectionectomy (right hepatic vein preserving) [33].
34. A two-month-old infant presented to the hospital for umbilical polypectomy. He was born at a gestational age of 38 + 1 weeks, weighing 2.99 kg, and had no particular features other than a mass protruding from the navel. After admission, vital signs were normal, and the weight was measured at 5.4 kg, and laboratory tests showed no abnormalities [34].
35. A 16-year-old male patient (height 179 cm, weight 60.8 kg) was admitted to the thoracic surgery department with a complaint of sudden chest pain that occurred two days ago. The patient had no medical history and was a never-smoker and the patient's American Society of Anesthesiologists physical status classification was 1 [35].
36. A 59-year-old female patient (height, 162 cm; weight, 59 kg) with type I HAE was scheduled for total laparoscopic hysterectomy [36].

37. A 34-year-old female patient (height, 168 cm; weight, 59 kg) was scheduled to undergo orthognathic surgery under general anesthesia for malocclusion and facial asymmetry [37].
38. A 47-year-old female (body weight 49.6 kg, height 159 cm) suffered from acute liver failure due to alcoholic hepatitis [38].
39. A 60-year-old man diagnosed with esophageal adenoma (high-grade dysplasia) was scheduled for ESD under general anesthesia. He was 169.9 cm tall and weighed 69.2 kg [39].
40. A 73-year-old patient with a height of 162.1 cm and weight of 71.4 kg was admitted to the hospital for scheduled transurethral resection of bladder tumor and the prostate (TUR-BT and TURP) for the treatment of bladder cancer and benign prostate hyperplasia [40].
41. A 75-year-old female (weight, 51 kg; height, 146 cm) was scheduled to undergo a decompressive lumbar laminectomy and interbody fusion (lumbar level, 3-4 and 4-5) under general anesthesia [41].
42. A 13-year-old male patient was scheduled for elective insertion of a continuous ambulatory peritoneal dialysis (CAPD) catheter. The admission height and weight were 135 cm and 31 kg, respectively [42].
43. A 75-year-old male weighing 50 kg and having a height of 165 cm was scheduled to undergo right upper lobectomy for the treatment of non-small cell lung cancer [43].
44. A 76-year-old female patient, 149 cm in height and 62 kg in weight, was scheduled for laparoscopic surgery on a calcified mass, 3.1 cm in size, located on the rectovesical pouch. She was also scheduled for a hysteroscopy due to intrauterine septae [44].
45. A 24-year-old man, with a height of 190 cm and weight of 52 kg, was admitted to our hospital with right neck of femur fracture to undergo closed reduction and internal fixation [45].
46. A 58-year-old male patient (165 cm/71.4 kg) was admitted for further evaluation and management of a submucosal lesion in the distal esophagus. The endoscopist planned enucleation of the leiomyoma by endoscopic submucosal tunnel resection (ESTR) and requested for general anesthesia for the procedure [46].
47. Our patient, a 67-year-old male weighing 66.8 kg and measuring 169.4 cm in height, was scheduled for sutureless aortic valve replacement to treat severe aortic stenosis [47].
48. A 59-year-old woman presented for living donor LT and as a liver donor for domino LT [48].
49. A 77-year-old woman (height 148.5 cm, weight 70 kg) was admitted to undergo posterior lumbar interbody fusion for lumbar spinal stenosis (L1–3) [49].

50. A 70-year-old male (170 cm, 59 kg) was admitted for a cystic mass (10.4 × 7.9 × 7.6 cm) of the liver and decided to undergo right hepatectomy [50].
51. An 83-year-old man (weight: 65 kg, height: 167 cm) was scheduled to undergo open reduction and internal fixation of a pertrochanteric fracture of the right femur [51].
52. An 18-year-old female (weight, 53 kg, height, 162 cm, and BMI, 20.2 kg/m2) with a history of Goldenhar syndrome presented for placement of a right mandibular distractor, right retroauricular dilator, and stage I transfer of a prefabricated expanded flap under general anesthesia [52].
53. Our subject is a 56-year-old male weighing 53 KG and 160 cm tall who had a history of multiple cholelithiasis, as well as multiple choledochotomies over the past 30 years. He was admitted to the hospital complaining of recurrent right upper abdominal pain over the span of one week and abdominal CT showed varying degrees of intrahepatic bile duct dilatation with multiple stones.Preoperatively diagnosed with the following: (1) intrahepatic bile duct stones with cholangitis; and (2) biliary cirrhosis.The treatment plan of an elective right hepatectomy with bile-intestinal anastomosis and reconstruction was decided [53].
54. A 54-year-old male was admitted to the hospital for left far-lateral craniotomy and microsurgical clipping of a left posterior inferior cerebellar artery (PICA) aneurysm [54].
55. A 70-year-old man (weight 62 kg, height 170 cm) undergoing radiation therapy and hormone therapy for prostate cancer was admitted to the hospital for macro hematuria and anorexia, thus an urgent transurethral electrocoagulation was ordered after blood transfusion [55].
56. A 14-year-old boy (height, 135cm; weight, 26kg) with Moebius syndrome was scheduled to undergo surgery to remove a mandibular cyst under general anesthesia [56].
57. A 55-year-old man (95kg, 170cm, BMI 32,9kg.m−2) was admitted for emergency placement of a JJ stent [57].
58. An 83-year-old man (weight 60 kg, height 165 cm) was admitted to our hospital for laser TURP due to recurrent prostate adenoma [58].
59. This case describes a middle-aged female who presented to the ambulatory surgery center for right sided hemithyroidectomy. The patient’s height, weight, and BMI are 155 cm, 78 kg, and 32.5, respectively. The patient was classified by the American Society of Anesthesiologists (ASA) as a risk of 3 [59].

60. A 66-year-old male patient had experienced epigastric pain for half a month. Upon CT examination, pancreatic tail neoplasm was observed. The surgeon planned to resect the pancreatic tail under general anesthesia. He weighed 66 kg and was American Society of Anesthesiologist classification II [60].

Table S2. Clinical text for a patient's medical history.

1. She had undergone surgery for diaphragmatic palsy 18 years prior [1].
2. The patient was diagnosed with hypertension but had no history of chest pain to suspect coronary disease [2].
3. Her medical history included well-controlled diabetes, Parkinson's disease, depression, anxiety, and dementia. She was taking valproate, olanzapine, quetiapine, donepezil, diazepam, levodopa, rasagiline, and pregabalin. Of these, only rasagiline was discontinued two weeks before surgery. Two years ago, she was admitted to a closed psychiatric ward after having experienced delusions and intermittent drowsiness for one year. Furthermore, she had undergone orthopedic surgery under general anesthesia five times in the last two years due to a distal humerus shaft fracture. No perioperative events occurred during the previous surgeries [3].
4. The patient was known to have rheumatoid arthritis and to be taking 15 mg of oral MTX per week for the previous five years, the last dose of which was taken two days prior to admission. A diagnostic cerebral angiogram was performed on admission with 50 ml of intravenous iohexol (Omnipaque, 350 mg iodine/ml, GE Healthcare Pvt. Ltd, India) before surgery [4].
5. She had a medical history of hypertension, hypothyroidism, depression, insomnia, and somatic symptom disorder and was diagnosed as having sick sinus syndrome nine months ago. A cardiologist had recommended that she needed a pacemaker implanted, but she rejected it. She was taking levothyroxine, antihypertensive agent, analgesics with several psychiatric drugs, including benzodiazepine, zolpidem, and pregabalin, and two more unidentified drugs prescribed by a local clinic [5].
6. The patient was a non-smoker with a history of hypertension, non-insulin-dependent diabetes mellitus on oral hypoglycemic agents, and epilepsy on sodium valproate. She had a surgical history of a laparoscopic cholecystectomy 11 years ago, which was uncomplicated according to the patient; however, no records were available for review. Additionally, the patient had a history of hyperthyroidism, had received radioactive iodine treatment more than 10 years prior, and was on oral levothyroxine replacement [6].
7. One year earlier, the patient developed acute pancreatitis and underwent mechanical ventilation by endotracheal intubation. After his discharge from the hospital for one month, a severe dyspnea

happened, caused by subglottic stenosis. His tracheal stenosis was mostly attributed to the previous instance of prolonged intubation, mechanical ventilation, and cuff injury [7].

8. The patient did not have any coexisting disease or history of seizure [8].
9. She had undergone uneventful surgery with general anesthesia for a laminectomy eight years earlier. She was taking oral hypoglycemic and antihypertensive agents with analgesics for back pain. She had no history of psychiatric illnesses or drug addiction [9].
10. She had known hypertension, type II diabetes mellitus, and obstructive sleep apnea [10].
11. Her previous medical history included diabetes mellitus and Parkinson's disease. She had been on insulin and levodopa [11].
12. He had amputation of the right index finger at another hospital 26 years ago, and had middle finger amputation and flap coverage in our orthopedic department seven years ago. Both surgeries were performed under general anesthesia without exposure to sugammadex. He had hypertension that was not treated. He did not have allergy history [12].
13. She had a history of cardiopulmonary resuscitation due to severe left ventricular dysfunction with an ejection fraction of 30% that was diagnosed as stress-induced cardiomyopathy caused by pneumonia a year ago. She also had history of congestive heart failure, controlled hypertension, and chronic obstructive lung disease. A recently performed transthoracic echocardiograph showed a left ventricular ejection fraction of 68%. Her pulmonary systolic arterial pressure was 41 mmHg without regional wall motion abnormality [13].
14. He had normal airway features: good mouth opening, Mallampati score of 1, thyromental distance > 6 cm and normal tongue protrusion [15].
15. no known comorbidities or history of previous surgeries [17].
16. His co-morbidities included asthma and hypertension. He had a Mallampati score of 3, large thick neck, and severe obstructive sleep apnea (OSA) [18].
17. He had well-controlled diabetes mellitus and reported no allergies except to cat hair. He had undergone general anesthesia 10 years prior, but it was uneventful [19].
18. The patient had a normal airway with no major risk factors [20].
19. He had a history of myocardial infarction one year previously, for which he had undergone insertion of multiple bare metal coronary artery stents. He had developed post-infarction dilated cardiomyopathy with a severe reduction in ejection fraction (30%), a marked increase in the left ventricular diastolic

diameter (6.8 cm), and global left ventricular hypokinesia with apical akinesia. He also had moderate mitral and minimal tricuspid valve insufficiency. His past medical history also included hypertension, diagnosed forty years ago. He was also a current, light smoker (less than 10 cigarettes per day)[23].

20. The patient had been diagnosed with CCHS based on clinical symptoms that emerged immediately after birth. At one month of age, the patient underwent a tracheostomy; at eight years of age, he underwent another surgery for the primary closure of this tracheostomy under general anesthesia. He was not admitted to the intensive care unit after the operation. He had no other previous history of anesthesia and surgery. The patient had strabismus, had fainted once previously, and had lived without restrictions in his daily life other than a few episodes of dizziness [24].
21. The patient had no problems with cognition or communication but complained of intermittent headaches with a visual analog score of 3–5 [25].
22. He had undergone percutaneous coronary intervention on the left anterior descending and circumflex branches 8 years prior because of angina pectoris with other complications such as diabetes, stroke, chronic atrial fibrillation, chronic renal failure, and spinal canal stenosis [26].
23. The child was previously diagnosed with pyruvate dehydrogenase E1-alpha deficiency two months after birth. At the time of diagnosis, the child's laboratory result showed lactic acid 6.4 mM/L. There were no abnormal findings in the parental phenotype, and the parental genetic test results were normal. Therefore, the patient had a de novo pathogenic variant of pyruvate dehydrogenase E1-alpha deficiency. He had a history of multiple admissions and discharges with similar events [27].
24. She had been on medication for 12 years after being diagnosed with hypertension and cerebral infarction. Her most recent medical history includes admission to the emergency room four weeks ago for a stuporous level of consciousness. She was sedated due to agitated behavior and intubated in the emergency room with 7.5-mm inner diameter (ID) and 10.0-mm outer diameter (OD) plain cuffed ETT (Rüsch® Super Safety ClearTM, Teleflex Medical) for ventilatory support [28].
25. The patient was diagnosed with subarachnoid hemorrhage and meningioma, and underwent emergency craniectomy and cerebral aneurysm clipping 3 weeks ago. The subarachnoid hemorrhage was caused by an aneurysmal rupture of the left posterior communicating artery [29].
26. Her underlying disease, SLE, was diagnosed at the age of 11 years (15 years before this admission). The diagnostic criteria related to SLE included autoimmune hemolytic anemia, lymphopenia, neutropenia, malar rash, oral ulcer, positive anti-double-stranded DNA, positive antinuclear antibody,

and lupus nephritis with proteinuria. The woman was initially treated with prednisolone and hydroxychloroquine for 6 years until she achieved clinical remission with successful tapering off of steroids. Until the age of 18 years (9 years ago), the sole symptom of SLE was her becoming easily fatigued [32].

27. The patient had experienced a cerebral infarction at 67 years of age but he did not present with any significant sequelae. He also had a history of benign prostatic hyperplasia and major depressive disorder [33].
28. The patient had no medical history and was a never-smoker [35].
29. The patient's medical history revealed a diagnosis of type I HAE at the age of 49 years, based on genetic testing. She experienced recurrent episodes of angioedema involving legs or hands once every 2–3 months. However, she had not experienced an edema attack for the past one year, so she had been using 100 mg of danazol once a week for long-term symptom control [36].
30. The patient had no medical history or specific findings other than orthodontic findings [37].
31. The patient was a heavy drinker and was hospitalized due to mental changes two months prior to LT. She received steroid treatment for severe alcoholic hepatitis, but it was tapered due to lack of response. Treatment for hepatic encephalopathy and endoscopic variceal obturation for gastric variceal bleeding was performed as well. One week before LT, she was intubated due to dyspnea caused by pulmonary edema, probably due to excessive transfusion and fluid treatment after hematemesis [38].
32. He had underlying hypertension, with normal preoperative laboratory and chest radiography findings. He had no history of pulmonary disease [39].
33. The patient had been taking metformin and rosuvastatin for type 2 diabetes mellitus and hyperlipidemia, respectively [40].
34. She had well-controlled hypertension. Five years earlier, she underwent an uneventful procedure under general anesthesia (transobturator vaginal tape operation). She reported no known allergy to medication, food, or latex [41].
35. The patient was born at 40 weeks by normal vaginal delivery and was admitted to the neonatal intensive care unit for 10 days because of sepsis. MD was diagnosed at 12 months of age and then JS was diagnosed during outpatient observation. The patient started hemodialysis due to end-stage renal disease at the age of 8 years and was hospitalized at age 10 years with intracranial hemorrhage due to high blood pressure. The patient was admitted at age 11 years for uncontrolled hypertension and a

change in the permanent catheter, but hyperkalemia-induced cardiac arrest occurred. The patient returned to spontaneous circulation after 1 h of cardiopulmonary re-suscitation (CPR) and was transferred to the intensive care unit (ICU) to receive post-CPR care for 2 weeks. Subsequently, the patient was hospitalized and discharged repeatedly due to heart, lung, and kidney problems. He underwent regular hemodialysis; however, hemoglobin level and blood pressure continued to fall and could not be controlled [42].

36. His significant medical history was chronic obstructive pulmonary disease (COPD) and was a 55-pack-year smoker [43].

37. The patient had a medical history of diabetes mellitus and hyperthyroidism. [44]

38 He was diagnosed with WHS after birth. Although he had slight mental retardation, he was able to ambulate and perform simple commands [45].

39. He was diagnosed and treated for pulmonary tuberculosis 25 years ago, which resulted in severe sequelae in both lungs, and also underwent surgical resection 14 years prior to treat abdominal liposarcoma. Additionally, our patient was hospitalized for treatment of atrial fibrillation and cardiac insufficiency 7 years ago and diagnosed with hypertension, diabetes, and chronic obstructive pulmonary disease 4 years ago. He was prescribed the following medications: rivaroxaban, digoxin, angiotensin receptor blocker, calcium channel blocker, and an oral hypoglycemic agent. However, rivaroxaban was interrupted 3 days before surgery [47].

40. She presented with symptoms of edema and paresthesia of both lower extremities 7 years ago and was found to have cardiomegaly on chest radiography. She was diagnosed with amyloidosis 1 year previously while undergoing a pericardial window operation for pericardial effusion [48].

41. She had hypertension that was treated with amlodipine (a calcium channel blocker) and carvedilol (a beta-blocker) [49].

42. The patient had a medical history of hypertension and diabetes mellitus [50].

43. His past medical history was nonspecific. He had no history of allergy to drugs or food [51].

44. The patient had no symptoms of respiratory disease, such as stridor, shortness of breathing, limitation on physical exertion, and no history of recurrent croup. No history of surgery and endotracheal intubation [52].

45. The patient had a history of well-controlled hypertension, was able to participate in 7–10 metabolic equivalents of task. He had no known adverse reactions to anesthetic agents in the past [54].

46. The patient was diagnosed with Moebius syndrome at the age of 7 years based on clinical symptoms, including facial paralysis, dysphagia, and incomplete quadriplegia since birth. Cerebral nerve palsy affected his trigeminal (V), facial (VII), glos- sopharyngeal (IX), vagus (X), and hypoglossal nerves (XII). He also had a severe intellectual disability; therefore, he needed assistance with all activities of daily living. He was intellectually impaired but was able to follow instructions such as making a peace sign to the camera, allowing the measurement of blood pressure, opening his mouth wide, and allowing the collection of blood samples (with physical restraint). However, he was incapable of engaging in meaningful verbal communication. He underwent gastrostomy at the age of one for lack of oral intake caused by dysphagia arising from cerebral nerve palsy. He often experienced aspiration pneumonia and was hospitalized for it at least once a year. He had a history of epilepsy but had not experienced epileptic seizures for more than 2 years at the time of admission [56].
47. The patient had cerebral palsy, intellectual disability, deafness, and left hemiparesis due to prematurity and neonatal distress. He had acquired chronic obstructive pulmonary disease (COPD) with recurrent bronchial infections, pulmonary aspiration, and bronchospasms. He also had sleep apnoea syndrome but was not adherent to continuous positive airway pressure (CPAP) treatment [57].
48. Past medical history included hypertension, peptic ulcer bleeding and C6–C7 disc herniation [58].
49. Pre-operatively, a full history and physical exam were obtained. The patient had received anesthesia in the past without complications. The patient is a non-smoker [59].
50. The patient developed hypertension [60].

Table 3S. Clinical text for a patient's clinical tests.

1. The patient's chest radiography and computed tomography revealed dextrocardia and bronchiectasis without situs inversus. In addition, patients had a tracheal bronchus of the apical segment in the RUL. Auscultation was normal, and the pulmonary function test showed a mild restrictive pattern [1].
2. The echocardiography showed normal wall motion and ejection fraction with concentric left ventricular hypertrophy and mild diastolic dysfunction [2].
3. Preoperative blood test results were unremarkable, and echocardiography revealed normal contractility, with an ejection fraction of 60% [4].
4. A preoperative electrocardiogram showed normal sinus rhythms and a heart rate of 78 beats/min. Transthoracic echocardiography showed an ejection fraction of 56% [5].
5. Routine tests for thoracic surgery showed normal spirometry (FEV1: 1.44 L [85% of the predicted value], FVC: 1.72 L [92% of the predicted value], and DLCO 94% of the predicted value). Transthoracic echocardiography revealed an ejection fraction of 66% and an absence of regional wall motion abnormalities. The patient had aortic valve sclerosis but no stenosis, and all other valves were normal [6].
6. A recently performed transthoracic echocardiograph showed a left ventricular ejection fraction of 68%. Her pulmonary systolic arterial pressure was 41 mmHg without regional wall motion abnormality.[13]
7. Preoperative laboratory findings, chest radiography, electrocardiography, and pulmonary function tests were normal [19].
8. His preoperative chest radiograph revealed cardiomegaly [23].
9. In 2012, a transthoracic echocardiograph and a right heart catheterization with a pulmonary vasodilatation test confirmed the diagnosis of PAH. The condition did not respond to nitric oxide (mPAP, 42.5 mmHg; pulmonary arterial pressure [PAP], 50/38 mmHg; left ventricular ejection fraction, 68%; D-shaped left ventricle, right ventricular dilatation) [32].
10. The patient's left ventricular systolic function was normal (visual estimated ejection fraction 60%), with no regional wall motion abnormality. Impaired relaxation and increased intima-media thickness of the right common carotid artery were observed on TTE [33].
11. Laboratory test and electrocardiogram (ECG) findings did not reveal any abnormalities [35].
12. Chest X-ray and electrocardiography findings were normal [36].
13. Echocardiography was normal [38].

14. with normal preoperative laboratory and chest radiography findings [39].

15. Results of preoperative laboratory examinations, chest radiography, electrocardiography, transthoracic echocardiography, thallium scan, and pulmonary function tests were normal [41].

16. The preoperative pulmonary function test showed a moderate obstructive pattern with a forced expiratory volume of 1.64 L (61% of predicted) in one second. The chest computerized tomography revealed subsegmental atelectasis in bilateral lower lobes and right middle lobe, emphysema with bullae in bilateral lungs, and scanty right pleural effusion. An electrocardiogram (ECG) performed before the surgery showed right bundle branch block, and transthoracic echocardiography revealed normal left ventricle (LV) global systolic function (ejection fraction [EF] = 58%) and abnormal relaxation of LV filling pattern [43].

17. No specific problems were noted on the transthoracic echocardiography, although ventricular premature beats were present on the preoperative electrocardiogram. chest X-ray did not show any abnormal findings [45].

18. no abnormal findings were observed in the chest radiograph, electrocardiogram, or laboratory tests [46].

19. The patient exhibited atrial fibrillation, premature ventricular contraction, left anterior fascicular block, and anterior infarction from electrocardiogram (ECG), with an aortic valve blood flow velocity of 4.3 m/s, mean pressure of 46 mmHg, and aortic valve surface area of 0.7 cm2 from echocardiography. A chest radiography showed pleural effusion in the right lung, an atypical pulmonary nodule, lung parenchymal distortion, bilateral upper lobe pleural thickening, and emphysema, while a pulmonary function test revealed FEV1/FVC was 40%, FEV1 was 27%, FVE was 47%, and TLC was 68%, indicating mixed respiratory failure [47].

20. Chest radiography was consistent with previous findings of cardiomegaly without active lung lesions. Sinus rhythm with atrial premature complexes and left ventricular hypertrophy were found in electrocardiogram. An echocardiogram showed increased left ventricle (LV) wall thickness, diastolic dysfunction (grade 2) with increased LV filling pressure, both atrial enlargement, and a moderate amount of pericardial effusion. The LV ejection fraction was 60.5% without regional wall motion abnormality [48].

21. Chest radiography, electrocardiogram (ECG), and physical examination were normal. [49]

22. Pre-operative transthoracic echocardiography revealed a left ventricular ejection fraction of 44% and severe hypokinesia of the left ventricle. Coronary angiography showed no significant stenosis. Pulmonary function tests demonstrated a restrictive ventilation defect [50].
23. The patient's preoperative chest radiograph showed a right-sided pleural effusion but no dyspnea or decreased pulse oximetry (SpO2) without oxygen administration [55].
24. Chest X-ray (CXR) revealed a normal cardiac silhouette and no pulmonary lesions. Electrocardiogram (ECG) showed normal sinus rhythm without left ventricular hypertrophy or left atrial enlargement [58].
25. his electrocardiogram showed sinus rhythm and high voltage in the left ventricle [60].

References

1. Hwang BY, Kwon JY, Kim E, Baik J, Kim HJ, Heo Y, et al. The EZ-Blocker® for one-lung ventilation in a patient with Kartagener syndrome and tracheal bronchus -a case report. Korean J Anesthesiol. 2023; 76: 67-71.

2. Boo KY, Park SH, Park SK, Na C, Kim HJ. Cardiac arrest due to coronary vasospasm after sugammadex administration -a case report. Korean J Anesthesiol. 2023; 76: 72-6.

3. Baek S, Heo MH, Kim KW, Lee SI, Kim KT, Park JS, et al. Catatonia associated with prolonged stupor after general anesthesia in a patient with multiple neuropsychiatric disorders -a case report. Korean J Anesthesiol. 2023; 76: 383-8.

4. Vaithialingam B, Muthuchellappan R, Sundaram M. Fatal multiorgan dysfunction following repeated iodinated radiocontrast injection in a patient receiving low-dose oral methotrexate -a case report. Korean J Anesthesiol. 2022; 75: 283-5.

5. Kim SH, Kim KM, Lim YH, Yoo BH, Cho J, Jun IJ. Psychogenic coma after general anesthesia with remimazolam and remifentanil -a case report. Korean J Anesthesiol. 2022; 75: 449-52.

6. Chua JH, Sim BLH, Theng TKP, Chew S. Obstructive fibrinous pseudomembrane tracheitis after double-lumen tube intubation -a case report. Korean J Anesthesiol. 2022; 75: 350-3.

7. Peng L, Wei W. Tube-in-tube airway management in a patient with Montgomery T-tube in situ -a case report. Korean J Anesthesiol. 2021; 74: 165-8.

8. Kumar A, Kumar A, Kumar N, Kumar A. Intraoperative refractory status epilepticus caused by propofol -a case report. Korean J Anesthesiol. 2021; 74: 70-2.

9. Kim KO, Lee T, Kim T. Non-convulsive status epilepticus in the immediate postoperative period following spine surgery -a case report. Korean J Anesthesiol. 2021; 74: 541-5.

10. Syal R, Kumar R, Chhabra S, Agha M. Is the mid-transverse process to pleura block a better technique for patients with obesity undergoing modified radical mastectomy? Korean J Anesthesiol. 2020; 73: 462-4.

11. Park JH, Kwon JY, Lee SE, Kim YH, Kim SH. Sudden hemodynamic collapse after prone positioning on a Jackson spinal table for spinal surgery. Korean J Anesthesiol. 2020; 73: 71-4.

12. Choi SC, Han S, Kwak J, Lee JY. Anaphylaxis induced by sugammadex and sugammadex-rocuronium complex -a case report. Korean J Anesthesiol. 2020; 73: 342-6.

13. Yoon HS, Yu BW, Kim YM, Lee JH, Koh WU, Yang HS. Serratus anterior plane block combined with monitored anesthesia care for surgery of lateral side of breast -a case report. Korean J Anesthesiol. 2019; 72: 500-3.

14. van de Pas JM, van der Woude MC, Belgers HJ, Hulsewé KW, de Loos ER. Bronchus perforation by EZ-BlockerTM endobronchial blocker during esophageal resection after neoadjuvant chemoradiation -a case report. Korean J Anesthesiol. 2019; 72: 184-7.

15. Tham LY, Beh ZY, Shariffuddin, II, Wang CY. Unilateral hypoglossal nerve palsy after the use of laryngeal mask airway (LMA) Protector. Korean J Anesthesiol. 2019; 72: 606-9.

16. Sriramka B, Rao PB. Smartphones in the operating theatre: a vice may be a boon. Korean J Anesthesiol. 2019; 72: 283-4.

17. Mishra N, Singh S, Elayat A, Kaushal A. Cardiac arrest in the prone position caused by central venous cannulation-induced cardiac tamponade. Korean J Anesthesiol. 2019; 72: 394-5.

18. Lim WY, Wong P. Awake supraglottic airway guided flexible bronchoscopic intubation in patients with anticipated difficult airways: a case series and narrative review. Korean J Anesthesiol. 2019; 72: 548-57.

19. Kim GH, Choi WS, Kim JE, Yun MJ, Koo MS, Kwon M, et al. Anaphylactic shock after sugammadex administration, induced by formation of a sugammadex-rocuronium complex -a case report. Korean J Anesthesiol. 2019; 72: 495-9.

20. Karippacheril JG, Philip A, Ashraf Y. Forced cough for witnessed extreme bradycardia in hip arthroplasty: a maneuver in extremis. Korean J Anesthesiol. 2019; 72: 279-80.

21. Hoffman CR, Van Hal M, Tariq R, George S. Complications of fluid overload during hysteroscopic surgery. Korean J Anesthesiol. 2019; 72: 387-8.

22. Elkoundi A, Bentalha A, Kettani SEE, Mosadik A, Koraichi AE. Erector spinae plane block for pediatric hip surgery -a case report. Korean J Anesthesiol. 2019; 72: 68-71.

23. De Cassai A, Bonvicini D, Ruol M, Correale C, Furnari M. Erector spinae plane block combined with a novel technique for selective brachial plexus block in breast cancer surgery -a case report. Korean J Anesthesiol. 2019; 72: 270-4.

24. Choi Y, Lee S, Lee J, Woo S. Spinal anesthesia and postoperative epidural analgesia in a patient with congenital central hypoventilation syndrome -a case report. Korean J Anesthesiol. 2019; 72: 375-80.

25. Lee WK, Kim H, Bae MI, Choi SH, Min KT. Accidental intracerebral injection and seizure during scalp nerve blocks for awake craniotomy in a previously craniotomized patient -a case report. Korean J Anesthesiol. 2018; 71: 483-5.

26. Yamamoto Y, Tanaka N, Kadoya Y, Umehara M, Suzuka T, Kawaguchi M. Bolus intertransverse process block and continuous erector spinae plane block for perioperative analgesic management of video-assisted thoracoscopic surgery - Three cases report. Anesth Pain Med (Seoul). 2023; 18: 198-203.

27. Lim WY, Lee HJ, Park EJ, Jeon S, Do W, Kim HJ, et al. Perioperative considerations of pyruvate dehydrogenase complex deficiency: a case report of two consecutive anesthesia. Anesth Pain Med (Seoul). 2023.

28. Ki S, Cho SB, Park S, Lee J. Management of unanticipated difficult airway in a patient with well-visualized vocal cords using video laryngoscopy - A case report. Anesth Pain Med (Seoul). 2023; 18: 204-9.

29. Heo HJ, Kim YY, Lee JH, Cho HG, Kim G. Severe pancytopenia and coagulopathy discovered during anesthesia after pre-anesthetic evaluation - A report of two cases. Anesth Pain Med (Seoul). 2023; 18: 92-6.

30. Sinha C, Kumar A, Kumar A, Kumari P, Agrawal P, Sk A. Two-level bilateral ultrasound-guided erector spinae plane block for kyphosis corrective surgery - A case report. Anesth Pain Med (Seoul). 2022; 17: 327-30.

31. Rosales AL, Aypa NS. Clavipectoral plane block as a sole anesthetic technique for clavicle surgery - A case report. Anesth Pain Med (Seoul). 2022; 17: 93-7.

32. Lertkovit S, Nivatpumin P. Anesthetic management of cesarean delivery of parturient with systemic lupus erythematosus associated with pulmonary arterial hypertension - A case report. Anesth Pain Med (Seoul). 2022; 17: 291-7.

33. Kim H, Lee J, Lee SG, Shim KS. Detection of paradoxical carbon dioxide gas embolism with opening of patent foramen ovale by perioperative transesophageal echocardiography during laparoscopic hepatectomy - A case report. Anesth Pain Med (Seoul). 2022; 17: 397-403.

34. Kim H, Cho J, Lee S, Lim Y, Yoo B. The use of sugammadex in an infant with prolonged neuromuscular blockade - A case report. Anesth Pain Med (Seoul). 2022; 17: 52-6.

35. Han W, Kim GS, Lee JM, Lim CM, Yang HS, Jeong CY, et al. Unexpected pulmonary edema and cardiac arrest following wedge resection of spontaneous pneumothorax -A case report. Anesth Pain Med (Seoul). 2022; 17: 298-303.

36. Bang YS, Cho J, Park C. An anesthetic experience of hereditary angioedema type I patient undertook total laparoscopic hysterectomy - A case report. Anesth Pain Med (Seoul). 2022; 17: 235-8.

37. Seo SH, Lee JM, Lee JJ, Ahn EJ, Choi GJ, Kang H. Repeated endotracheal tube cuff tears during nasotracheal intubation due to nasal cavity orthodontic micro-implant - A case report. Anesth Pain Med (Seoul). 2021; 16: 398-402.

38. Oh Y, Yoo SY, Choi GS, Kim G. Liver transplantation of a patient with extreme thrombocytopenia - A case report. Anesth Pain Med (Seoul). 2021; 16: 279-83.

39. Oh SK, Cho SI, Won YJ, Yun JH. Bilateral tension pneumothorax during endoscopic submucosal dissection under general anesthesia diagnosed by point-of-care ultrasound - A case report. Anesth Pain Med (Seoul). 2021; 16: 171-6.

40. Kim SY, Lim Y, Bae GH, Suh DH, Kim KM. Numbness of the lower lip following urological surgery under general anesthesia - A case report. Anesth Pain Med (Seoul). 2021; 16: 201-4.

41. Kim SM, Oh SH, Ryu SA. Treatment of rocuronium-induced anaphylaxis using sugammadex - A case report. Anesth Pain Med (Seoul). 2021; 16: 56-9.

42. Kim JY, Jeong K, Han KS, Park JE, Kim MG, Jun MR. Anesthetic considerations of Joubert syndrome in patients with mitochondrial disease - A case report. Anesth Pain Med (Seoul). 2021; 16: 158-62.

43. Lee SK, Seo KH, Kim YJ, Youn EJ, Lee JS, Park J, et al. Cardiac arrest caused by contralateral tension pneumothorax during one-lung ventilation: - A case report. Anesth Pain Med (Seoul). 2020; 15: 78-82.

44. Lee EB, Park J, Lim HK, Kim YI, Jin Y, Lee KH. Complications of fluid overload during hysteroscopic surgery: cardiomyopathy and epistaxis - A case report. Anesth Pain Med (Seoul). 2020; 15: 61-5.

45. Kim HJ, You JA, Park S, Kim EJ, Park SJ, Kim HY. Anesthetic considerations for an adult with Wolf-Hirschhorn syndrome - A case report. Anesth Pain Med (Seoul). 2020; 15: 120-3.

46. Heo HJ, Lee JH, Kim YY, Baek SM, Kim KM, Jung DW. Invisible perforation during an endoscopic procedure of the esophagus under general anesthesia - A case report. Anesth Pain Med (Seoul). 2020; 15: 383-7.

47. Han I, Cho YW, Park SE, An MG, Kang HJ, Lee AR. Acute normovolemic hemodilution for a patient with secondary polycythemia undergoing aortic valve replacement due to severe aortic stenosis - A case report. Anesth Pain Med (Seoul). 2020; 15: 181-6.

48. Cha S, Kim J, Choi SJ, Kim GS. Domino living donor liver transplantation of familial amyloid polyneuropathy patient - A case report. Anesth Pain Med (Seoul). 2020; 15: 472-7.

49. Oh ID, Shin E, Jeon JM, Woo H, Choi JH. Use of methylene blue in vasoplegic syndrome that developed during non-cardiac surgery - A case report. Anesth Pain Med (Seoul). 2019; 14: 460-4.

50. Kim JH, Park S, Kim JY. Development of alveolar-pleural fistula during hepatectomy - A case report. Anesth Pain Med (Seoul). 2019; 14: 456-9.

51. Kim G, Kim G, Kwon M, Koo M, Yun M. Anaphylactic reaction with hydroxyethyl starch during anesthesia - A case report. Anesth Pain Med (Seoul). 2019; 14: 412-5.

52. Xing F, Deng XM, Yang D. Goldenhar syndrome complicated with subglottic airway stenosis: a case report. BMC Anesthesiol. 2023; 23: 210.

53. Chen Y, Liao Y, Chen X, Fan H, Lin D, Zheng T, et al. Recognition and management of hemorrhaging in combination with emerging enterogenic sepsis during a hepatectomy: a case report. BMC Anesthesiol. 2023; 23: 186.

54. Bassi JS, Hsu F, Mnatsakanyan L, Rajan GR. Acute airway obstruction requiring nasotracheal intubation following hypoglossal neuromonitoring: a case report. BMC Anesthesiol. 2023; 23: 149.

55. Sunabe M, Hoshi T, Niisato E. Respiratory distress associated with acute hydrothorax during transurethral electrocoagulation: a case report. BMC Anesthesiol. 2022; 22: 37.

56. Oda A, Oue K, Oda Y, Taguchi S, Takahashi T, Mukai A, et al. Difficult intubation and postoperative aspiration pneumonia associated with Moebius syndrome: a case report. BMC Anesthesiol. 2022; 22: 316.

57. Gaggini G, Nkamicaniye LM, Meyer S, Dubois PE. Expiratory central airway collapse during positive pressure ventilation: a case report. BMC Anesthesiol. 2022; 22: 50.

58. Chou CJ, Lai YC, Ou SY, Chen CH. Unexpected systolic anterior motion of the mitral valve-related hypoxemia during transurethral resection of the prostate under spinal anesthesia: a case report. BMC Anesthesiol. 2022; 22: 207.

59. Carpenter E, Norris L, Beniamin M. Adverse event using Medtronic NIM(™) EMG endotracheal tube on a patient receiving anesthesia for hemithyroidectomy: a case report. BMC Anesthesiol. 2022; 22: 220.

60. Zhou H, Pan Y, Liu C, Sun X. Emergence agitation after intraoperative neurolytic celiac plexus block with alcohol: a case report. BMC Anesthesiol. 2021; 21: 204.
